# Report of the Physiology and Pathology of the Blood

**Published:** 1846-11

**Authors:** 


### ECLECTIC DEPARTMENT,

### AND SPIRIT OF THE MEDICAL PERIODICAL PRESS.

---

### *Report of the Physiology and Pathology of the Blood.*

(Continued from page 289.)

We now pass to the consideration of another, perhaps the most important class of diseases—the phlegmasiæ,—which, though characterized by general febrile excitement like the pyrexiæ, present at the same time, a directly opposite condition of the blood.

Meckel has defined inflammation to be "congestion with a tendency to new production." This definition is, probably, as nearly correct as any that can be given; for from the very beginning to the termination of every inflammatory affection, there is a constant tendency in the blood, in proportion to the extent and severity of the disease, to the production and increase of its fibrinous element. The augmentation of the quantity of fibrine is so constant a sign of inflammation, that if we find more than five parts in 1000 in the course of any disease, we may positively affirm that some local inflammation exists.

During the active stage of inflammatory diseases, blood drawn from the patient presents all the characteristics indicative of an increase in the amount of fibrine. The coagulum is small, but firm, and comparatively free from serum, presenting, in most cases, when properly drawn, a well marked buffy coat, with a concave, or cupped surface. The buffy coat then; excepting in anæmic conditions of the blood, where the amount of fibrine, not being actually increased, is large only as compared with the diminished proportion of globules; always indicates an increase in the fibrine, and consequently the existence of some inflammatory affection.

The condition of the system, at the time of an inflammatory attack, seems not to influence the consequent increase of fibrine. A person may be exhausted by chronic disease, or present a confirmed anæmic condition, yet, upon the aucession of an inflammatory affection, there is in these, as in other cases, as constantly an increase of this constituent. In chlorotic parents, for instance, who have been attacked by acute articular rheumatism, bronchitis, pneumonia or erysipelas, the blood has been found to contain 6, 7, and even 8 parts of fibrine; and in cases of the pyrexiæ even, such as typhoid, typhus and miasmatic fevers, causing a diminution of fibrine, if, in the course of the disease, there supervenes an inflammatory affection, the amount of this element is always increased; thus presenting two forces acting upon the blood, one to diminish, the other to increase the proportion of this, its spontaneously coagulable element.

We may mention in this connection a curious fact, established by Gavarret, that the blood of animals, dying of hunger, shows an increase in the amount of fibrine, an effect, as appears from examinations, of inflammation of the coats of the stomach.

As to the comparative increase of fibrine, in inflammation of different tissues, it may be remarked, that of all diseases, pneumonia and acute articular rheumatism, are those in which the augmentation of this element is the greatest; the amount of fibrine in these affections often rising to the proportion of 10 in 1000.

In slight inflammation of the mucous membranes, unattended by febrile excitement, there is little or no appreciable change in the blood; but whenever the local disease becomes sufficiently intense to produce general reaction, the proportion of fibrine is found simultaneously to increase. It may in truth be stated, as a general fact, that independent of the organ affected, the accession and continuance of the accompanying fever bear a close relation to the constantly progressive changes in the sanguineous fluid. It seems not unreasonable to suppose, then, that the blood, circulating as it does through every organ and tissue of the body, thus surcharged with an undue proportion of its fibrinous element, produces that morbidly excited condition of every part, termed sympathetic fever.

The blood of individuals affected with mercurial ptyalism, presents always an increase of its fibrine, in proportion to the extent and severity of the inflammation. To illustrate, we give the following brief synopsis of three cases recorded by Andral. In the first case, profuse salivation was produced by 18.5 grs. calomel, augmenting the proportion of fibrine in the blood to 4.5. In the second case 9.5 grs. of calomel gave rise to very acute inflammatory action, increasing the fibrine to 5; and in the third case, more violent inflammation was produced than in either of the preceding, by the combined internal and external use of mercurials, raising the proportion of fibrine to 8.4.

Thus we see, that inflammation produced by mercury, appears not to differ in its effects from that produced by other causes. This fact may lead some to doubt, with good reason, the propriety of producing salivation in acute inflammatory diseases, and thus adding to the amount of fibrine already too abundant. It seems not, however, to contraindicate the more moderate use of the mineral, for it appears that the inflammation, not the medicine, caused the morbid change.

On the other hand, there seems to be some slight indication in the above fact, for the production of ptyalism to restore fibrine, and thus supply the deficiency in the pyrexiæ, such as typhus and typhoid fever.

Mercury is a remedy which has been, and is still used and recommended by practitioners, empirically, as it would seem, unless the above is a good reason for its use. Let us not, however, form hasty conclusions upon this point. In our contentions with disease, plausible reasons may justify the trial of a remedy, but favorable effects only recommend its general use.

Inflammations of the skin, from severe burns, erysipelas, blisters, &c., give rise also, to augmentation of fibrine in the blood. This fact suggests the probability, that the consequent increase of this element in cutaneous inflammatory affections, may be the principal and perhaps only reason, why blisters and other severe external irritants increase the sympathetic fever, and thus act unfavorably in the acute stages of these diseases. It may be true also, that the well known good effects of these remedies, in fevers of a typhoid type, and in old chronic affections, are, in part, the immediate results of an increase of fibrine, consequent upon the inflammatory action thus artificially produced.

It seems then, that inflammation, whatever may be the causes and circumstances attending its accession and continuance, appears and advances in most cases, simultaneously with the production and increase of this constituent of the circulating fluid.

As an exception, however, to the general rule, that increase of fibrine is accompanied with inflammatory action, it may be remarked, that pregnant women, during the last months of gestation, present blood, containing this element in a much higher proportion than the normal standard, varying for instance, from 4 to 5 in 1000 parts. It seems then, that "the blood on these occasions shows a remarkable tendency to assume the character of inflammatory blood; and assuredly there is matter for reflection in the relation which may exist between the modification then affected in the blood, and the development of those peculiar attacks, generally of an inflammatory aspect, to which women in child-bed are so liable."

As to the treatment of inflammation, it may be remarked, that the condition of the blood in these diseases seems to indicate the employment of such means as tend most directly to diminish the amount of its fibrinous element. Unfortunately however, no agent is known to the profession by means of which we can produce this effect promptly, and to such an extent as to give us unmistakable evidence for, or against this apparently legitimate conclusion.

Venesection has been found, by experience, to be one of the most effectual means of moderating and checking the progress of the phlegmasiæ. The most obvious effect of blood-letting, is that of abstracting a comparatively large proportion of the blood's most stimulating element, the globules. It acts favorably also by lessening, in common with the other ingredients, the exuberant fibrine, and thus removing, to some extent, the apparent cause of febrile action; and lastly, by diminishing the amount of blood circulating and thus favoring the absorption of fluids, it causes a dilution, and consequently, a more general diffusion of all its constituents. Cathartics, low diet, and most others of the class of antiphlogistic remedies, produce their beneficial effects, very probably, by diluting and diminishing the active constituents of the blood, and thus rendering this fluid less stimulating to the system generally, and to inflamed organs in particular.

Since the establishment of the important truth, that excess of coagulable matter is a distinguishing characteristic of inflammatory blood, it is said that well marked favorable results have been obtained from the administration of remedies, such as alkalies and other chemical agents, the well known effect of which, is to retard the coagulation of blood, and, as is believed by many, to prevent the rapid formation of fibrine. Nitrate of potash, for example, is a well known effectual remedy in this class of diseases, which, as it prevents the coagulation of blood after it is drawn, and even in the vessels, when injected into the veins, may depend, in part, for its beneficial effects in disease, upon this property.

In concluding these remarks upon the phenomena coincident with a pathological condition of the circulating fluid with regard to the fibrine, it may be stated, that among the most important of these, is a spontaneous tendency to loss of blood—the concomitant always to a deficiency of the coagulable as compared with its globular element.

It appears from facts before stated, that this want of normal proportions between these constituents is present in two apparently opposite conditions of the system — the plethoric and typhoid — in each of which, the blood presents the peculiar characteristics of excess of globules in the former, and deficiency of fibrine in the latter condition. Hence it is, that hemorrhage may be either active or passive, according as it occurs in one or the other of these abnormal conditions of the circulating fluid.

In plethoric persons, bleeding at the nose and other hemorrhages, if not profuse, or in such locations as to interfere with the vital functions, often relieve the unpleasant symptoms to which, as before stated, these individuals are sometimes subject. This is because a bleeding or hemorrhage, if not carried beyond a certain point, or excessive, diminishes the globules proportionally more than the fibrine, and thus for a time, removes the plethoric condition; but if, on the other hand, the abstraction of blood be carried too far, or if the active hemorrhage continue long and profuse, the fibrine begins to diminish at first, to the same, and afterwards, to a greater extent even than the globules, thus presenting at length, a condition of the blood, in which there is a deficiency of fibrine as compared with the globules, and thus changing its condition from that productive of a comparatively harmless escape of excess of globules, to that coincident with the more obstinate and dangerous disease, a passive hemorrhage.

From remarks, which have preceded upon this subject, it appears that the condition of the blood, favorable to passive hemorrhage, is present in the advanced stages of all the most malignant pyrexiæ, and as an effect of this morbid condition we have in these diseases, effusion of blood into the tissue of organs and under the cuticle, producing engorgement and petechial spots and patches; hemorrhages both internal and external; collections of blood in the cavities, and frequent bleeding from the gums, nose, rectum and other parts.

Thus we see that a loss of the fibrine of the blood is one of the most disastrous in its effects of all its pathological changes, a truth calling loudly upon the profession for some means of preventing the destruction of this important element.

No remedy is known, however, of sufficient activity and promptness in its effects, to modify this condition of the blood, to any appreciable extent, in the most malignant forms of this class of affections. It is evident, then, that our attention, for the present, should be directed to the first stages, and milder forms of these diseases, and to the nature and mode of action of those remedies which have been found more effectual in these more simple cases, where their effects can be seen and appreciated.

In all of the more chronic and milder forms of this class of diseases, with blood deficient in fibrine, such as scurvy, &c., more or less, of the following symptoms, always present themselves: pale, livid and dusky countenances, petechiæ, ecchymoses, soft, swollen and bleeding gums, debility, general numbness, heaviness of the head and vertigo. Blood drawn from an individual with such symptoms, coagulates slowly and imperfectly, the clot is, generally, large but soft, and often in separate fragments, never however presenting the buffy coat.

As to the therapeutic agents to be employed in diseases of this class, it may be remarked, that the causes, giving rise to them, as well as certain attendant conditions of the blood, give us some important indications.

The causes which most commonly give rise to this abnormal condition of the blood, are a damp and vitiated atmosphere, insufficient and improper food, such as that deficient in albumen and fibrine, the excessive use in food or otherwise of neutral salts or alkaline substances, such as common salt, salæratus, &c. Of the bad effects of the last named class of chemical substances, we have ample evidence in the condition of the blood coincident with a loss of fibrine.

It is true that the loss of a large proportion of the blood's coagulable element in these diseases, is the change made most appreciable by chemical analysis. "But is this the ultimate alteration we are permitted to arrive at? Before the fibrine decreases, has there not been some other change of composition in the blood, of which the depression of fibrine below its normal average is itself only the consequence? Upon this subject some facts may be cited. It may be remarked, that on throwing into the veins of living animals, a concentrated solution of subcarbonate of soda, an almost fluid blood was found in the bodies of these animals when dead, and that during life, their symptoms were analogous to those observed in diseases, in which the older writers admitted a state of dissolution of the blood. It may also be remarked that some authors declare, that they have found an excess of alkaline matters in the imperfectly coagulated blood of persons who died of low fevers or scurvy."* It is also true that blood drawn from patients with scurvy and other diseases, depending upon the same cause has presented the same characteristic.

It would appear then, that the treatment indicated in such cases, is to counteract the influence of the above named causes, by introducing in their place, an opposite train of influences, such as exercise, uncontaminated air, a plentiful supply of nutritious, nitrogenized food, and the displacement of alkaline ingesta by acid substances.

That the acids act favorably in these diseases, by neutralizing the excess of alkaline constituents in the blood, and favoring its coagulable tendency, is a view of their modus operandi of recent date. Whatever may be their mode of action, however, there can be no doubt with regard to their utility, or of the good effects of the general treatment above described, numerous results and long experience having established its efficiency.

In conclusion, it may be remarked that deficiency of albumen, is another very important alteration, not yet referred to, in the relative proportions of the blood's constituents, concomitant, always, with a tendency to dropsical effusions. Want of space, however, compels us to defer for the present, a consideration of this part of the subject. We hope, however, that the preceding partial exposition of some of the more modern, but generally adopted views, with regard to the composition and different conditions of the blood, imperfect as it is, and too limited for an exposition of the relation of facts referred to, may serve to direct attention to the importance of the subject and to the interesting facts and conclusions, which several distinguished men, now engaged in its investigation, are, from time to time, communicating to the profession. W. B. H.

